# Pipeline therapies for neovascular age related macular degeneration

**DOI:** 10.1186/s40942-021-00325-5

**Published:** 2021-10-01

**Authors:** Sruthi Arepalli, Peter K. Kaiser

**Affiliations:** Cole Eye Institute, Cleveland Clinic, Cleveland, OH USA

**Keywords:** Age related macular degeneration (AMD), Biosimilars, VEGF-A, PDGF, Tie-2

## Abstract

Age related macular degeneration (AMD) is the most common cause of vision loss in the elderly population. Neovascular AMD comprises 10% of all cases and can lead to devastating visual loss due to choroidal neovascularization (CNV). There are various cytokine pathways involved in the formation and leakage from CNV. Prior treatments have included focal laser therapy, verteporfin (Visudyne, Bausch and Lomb, Rochester, New York) ocular photodynamic therapy, transpupillary thermotherapy, intravitreal steroids and surgical excision of choroidal neovascular membranes. Currently, the major therapies in AMD focus on the VEGF-A pathway, of which the most common are bevacizumab (Avastin; Genentech, San Francisco, California), ranibizumab (Lucentis; Genentech, South San Francisco, California), and aflibercept (Eylea; Regeneron, Tarrytown, New York). Anti-VEGF agents have revolutionized our treatment of wet AMD; however, real world studies have shown limited visual improvement in patients over time, largely due to the large treatment burden. Cheaper alternatives, including ranibizumab biosimilars, include razumab (Intas Pharmaceuticals Ltd., Ahmedabad, India), FYB 201 (Formycon AG, Munich, Germany and Bioeq Gmbh Holzkirchen, Germany), SB-11 (Samsung Bioepsis, Incheon, South Korea), xlucane (Xbrane Biopharma, Solna, Sweden), PF582 (Pfnex, San Diego, California), CHS3551 (Coherus BioSciences, Redwood City, California). Additionally, aflibercept biosimilars under development include FYB203 (Formycon AG, Munich, Germany and Bioeq Gmbh Holzkirchen, Germany), ALT-L9 (Alteogen, Deajeon, South Korea), MYL1710 (Momenta Pharamaceuticals, Cambridge, MA, and Mylan Pharmacueticals, Canonsburg, PA), CHS-2020 (Coherus BioSciences, Redwood City, California). Those in the pipeline of VEGF targets include abicipar pegol (Abicipar; Allergan, Coolock, Dublin), OPT-302 (Opthea; OPTHEA limited; Victoria, Melbourne), conbercept (Lumitin; Chengdu Kanghong Pharmaceutical Group, Chengdu, Sichuan), and KSI-301 (Kodiak Sciences, Palo Alto, CA). There are also combination medications, which target VEGF and PDGF, VEGF and tissue factor, VEGF and Tie-2, which this paper will also discuss in depth. Furthermore, long lasting depots, such as the ranibizumab port delivery system (PDS) (Genentech, San Francisco, CA), as well as others are under evaluation. Gene therapy present possible longer treatments options as well and are reviewed here. This paper will highlight the past approved medications as well as pipeline therapies for neovascular AMD.

## Background

In the western world, the late forms of age-related macular degeneration (AMD) are the most common cause of irreversible vision loss in patients 65 year or older, with an anticipated rise to 288 million cases worldwide [1, 2]. AMD is divided into two subsets: non-neovascular or dry and neovascular or wet, with the former comprising the majority of cases (90%) [3, 4]. The non-neovascular variant usually results in steady degeneration of the outer retina and eventually geographic atrophy with gradual visual decline. In contrast, neovascular AMD is characterized by choroidal neovascular membrane formation, exudation and fibrosis leading to acute visual loss [5]. A major cytokine involved in the development of neovascular membrane formation is VEGF-A [6]. This cytokine has served as a therapeutic target for many neovascular AMD therapies [7].

While many trials have shown beneficial results with anti-VEGF medications, there are limitations to their use. Previous studies have shown that 20% of patients still lose vision and half ultimately do not obtain 20/40 vision [8, 9]. Additionally, the real-world application of these medications has shown that the visual outcomes often fall short of those in prior published randomized clinical trials. This may be secondary to poor compliance, lower injection rates, and under treatment of disease [10–13]. Therefore, an interest remains in targeting other molecules involved in the angiogenic process, as well as longer lasting medications to improve patient compliance and treatment rates. This review aims to review the existing treatments for neovascular AMD, as well pipeline therapies, including biosimilar alternatives, new intravitreal agents, sustained delivery systems and gene therapy.

## Main text

### Prior treatments

Multiple prior treatments for neovascular AMD have fallen out of favor, as they did not improve visual potential including focal laser therapy, verteporfin (Visudyne, Bausch and Lomb, Rochester, New York) ocular photodynamic therapy, transpupillary thermotherapy, focal radiation treatment, intravitreal steroid administration and surgical excision of choroidal neovascular membranes [14].

### Current treatments

Currently, all available treatments center on the inhibiting the VEGF pathway. Five treatments are currently available and listed in chronological order of introduction.

#### Pegaptanib

Approved in 2004, pegaptanib sodium (Macugen; Bausch and Lomb, Rochester, New York) is a pegylated aptamer that targets only the 165 isoform of VEGF-A. This molecule showed a reduction in vision loss compared to those treated with sham in neovascular AMD when given as an intravitreal injection at 6 week intervals [15]. As more effective anti-VEGF treatments have entered the treatment market, the use of this medication has been greatly reduced.

#### Bevacizumab

Bevacizumab (Avastin; Genentech, San Francisco, California) is full-length, humanized, recombinant monoclonal antibody against all isoforms of VEGF-A. This medication has been approved in the oncology realm for the treatment of metastatic colon cancer as an intravenous medication but has been used off-label as an intravitreal injection since 2005 for neovascular AMD when the results of the ranibizumab pivotal studies were known but the drug was not yet available [7, 16]. Due to its significantly lower cost since each vial of bevacizumab can be fractionated into numerous smaller doses for ocular use, it is often used as a first line agent and has been shown to be non-inferior to ranibizumab in various comparative studies in terms of both efficacy and safety [17].

#### Ranibizumab

Ranibizumab (Lucentis; Genentech, South San Francisco, California) is a affinity-matured, humanized, monoclonal antibody fragment that targets all isoforms of VEGF-A [18]. It was developed specifically for ocular use at the same time as bevacizumab at Genentech and the Fab fragment was chosen to better penetrate the retina. Approved in 2006, it has shown improvement in vision as compared to sham in the MARINA study and to verteporfin ocular photodynamic therapy in predominantly classic lesions in the ANCHOR study [8, 19]. These pivotal studies were the first to show on average patients gained vision after anti-VEGF therapy (Table 1).

**Table 1:** Currently available anti-VEGF therapies

Generic	Brand name, manufacturer	Target/mechanism
Pegaptanib sodium	Macugen; Bausch and Lomb, Rochester, New York	165 isoform of VEGF-A
Bevacizumab	Avastin; Genentech, San Francisco, California	All isoforms of VEGF-A
Ranibizumab	Lucentis; Genentech, South San Francisco, California	All isoforms of VEGF-A
Aflibercept	Eylea; Regeneron, Tarrytown, New York	VEGF-A and B, placental growth factor (PGF)
Brolucizumab	Beovu; Novartis, Logo, Basel	VEGF-A

#### Aflibercept

Aflibercept (Eylea; Regeneron, Tarrytown, New York), approved in 2011, is a recombinant fusion protein of specific domains from human VEGF receptor 1 and 2 combined with the Fc from human IgG1, allowing it to target VEGF-A and B, as well as placental growth factor (PGF) [7]. It has been shown to be non-inferior to monthly ranibizumab in the VIEW-1 and VIEW-2 studies, where aflibercept was administered monthly for 3 months and then transitioned to every 2 months over a 2 year period [20]. This study, along with others, helped show that the medication can be dosed at a longer dosing interval of every 8 weeks [9].

#### Brolucizumab

Brolucizumab (Beovu; Novartis, Logo, Basel) was approved in 2019. It is a single chain antibody fragment, which also targets VEGF-A [21]. Given its smaller size, it allows for a higher molar concentration in a 0.05-ml (ml) injection, which may provide better treatment duration [7]. The HAWK and HARRIER trials evaluated the medication at 12-week intervals in the absence of disease activity with a drop to q8 week dosing if disease activity was seen compared to fixed 8-week dosing for aflibercept [22, 23]. In the phase 3 studies, brolucizumab was shown to be non-inferior to aflibercept. Additionally, those treated with brolucizumab displayed a significant difference in the reduction of retinal fluid and central subfield thickness [23]. Brolucizmab maintained a significant number of patients on 12-week interval treatment periods, with over 50% of patients at the 48-week period in HAWK and HARRIER. Over 75% of those patients were maintained at the 12-week interval at 96 weeks [23]. While promising and effective, there have been some concerns surrounding the inflammatory profile of the drug. Recent reports have discussed the occurrence of intraocular inflammation, including retinal vasculitis and retinal occlusive vasculitis, which may limit its use [24].

## Drugs in the pipeline

### Biosimilars

The previously mentioned treatments have provided stabilization of the disease process in neovascular AMD. However, the regimented schedule of injections creates a financial and social burden which may lower patients’ long-term compliance. Prior studies have shown that inconsistent treatment is linked to lower visual acuity outcomes [11]. In order to decrease the financial demands of injections, pharmaceutical companies have attempted to create biosimilar medications as a cheaper treatment alternative since the patents for ranibizumab and bevacizumab have lapsed in the US [25]. It is important to note that biosimilar medications are not the same as generic medications; they attempt to replicate the therapeutic endpoint of existing medications rather than copy their molecular structure [26]. The requirements for FDA approval differs between generic and biosimilar medications. Interestingly when a biosimilar is approved in one indication it gains approval for all approved indications of the reference product (Table 2).

**Table 2:** Neovascular age related macular degeneration therapies in the pipeline

Class	Brand name, manufacturer	Target/mechanism	Status
Biosimilars	Razumab (Intas Pharmaceuticals Ltd., Ahmedabad, India)	Ranibizumab biosimilar	Approved
	FYB 201 (Formycon AG, Munich, Germany and Bioeq Gmbh Holzkirchen, Germany)	Ranibizumab biosimilar	Phase III trial completed
	SB-11 (Samsung Bioepsis, Incheon, South Korea)	Ranibizumab biosimilar	Phase III trial completed
	Xlucane (Xbrane Biopharma, Solna, Sweden)	Ranibizumab biosimilar	Phase III trial underway
	PF582 (Pfnex, San Diego, California)	Ranibizumab biosimilar	Phase I/II trial completed
	CHS3551 (Coherus BioSciences, Redwood City, California)	Ranibizumab biosimilar	Pre-clinical investigation
	FYB203 (Formycon AG, Munich, Germany and Bioeq Gmbh Holzkirchen, Germany)	Aflibercept biosimilar	Pre-clinical investigation
	ALT-L9 (Alteogen, Deajeon, South Korea)	Aflibercept biosimilar	Entering phase I trial
	MYL1710 (Momenta Pharamaceuticals, Cambridge, MA, and Mylan Pharmacueticals, Canonsburg, PA)	Aflibercept biosimilar	Phase III trial underway
	CHS-2020 (Coherus BioSciences, Redwood City, California)	Aflibercept biosimilar	Pre-clinical investigation
	ONS-5010 (Lytenava, Outlook Therapeutics, Cranberry Township, New Jersey)	Bevacizumab biosimilar	Phase III trial completed
Anti-VEGF	Abicipar pegol (Abicipar; Allergan, Coolock, Dublin)	VEGF-A	Phase III trial completed
	OPT-302 (Opthea; OPTHEA limited; Victoria, Melbourne)	VEGF-C and VEGF-D	Phase IIb trial completed
	Conbercept (Lumitin; Chengdu Kanghong Pharmaceutical Group, Chengdu, Sichuan)	VEGF-A, VEGF-B, and PGF	Phase III trial underway
	KSI-301 (Kodiak Sciences, Palo Alto, CA)	VEGF-A	Phase II trial underway
Anti- PDGF	CLS-AX (Ataxinib; Clearside Biomedical, Alpharetta, Georgia)	VEGF and PDGF	Pre-clinical investigation
	DE-120 (Santen Pharmaceuticals, Kita-Ku, Osaka)	VEGF-A and PDGF	Pre-clinical investigation halted
	Pegpleranib (Fovista; Ophthotech, Princeton, New Jersey)	PDGF-BB and PDGF-AB	Phase III trial completed
	Rinucumab (Regeneron, Tarrytown, New York)	PDGF-B	Phase II trial completed
	X-82 (Tyrogenex, Rockville, Maryland)	VEGF-A and PDGF	Phase II trial completed
Anti-tissue factor (TF)	ICON-1 (Iconic Therapeutics, San Francisco, California)	TF and natural killer cells	Phase II trial completed
Tie-2 receptor	Faricimab (Roche/Genentech, San Francisco, CA)	VEGF-A and Ang-2	Phase II trial completed
	REGN910 (Nevascumab; Regeneron, Tarrytown, New York)	Ang-2	Phase II trial completed
	ARP-1536 (Aerpio Therapeutics, Blue Ash, Ohio)	VE-PTP	Pre-clinical investigation
	DE-122 (Carotuximab; Santen, Kita-Ku, Osaka, and TRACON Pharmaceuticals, San Diego, CA)	Endoglin	Phase II trial completed
	AXT-107 (AsclepiX Therapeutics, Jersey City, New Jersey), inhibits both	VEGFR2	Pre-clinical investigation
Sustained treatments	Ranibizumab PDS (Genentech, San Francisco, CA)	VEGF-A	Phase III completed
	GB-102 (Sunitinib maleate; GrayBug Vision, Redwood City, CA)	VEGF-A and PDGF	Phase II underway
	OTX-TKI, a hydrogel depot of the tyrosine kinase inhibitor, sunitinib (Ocular Therapeutix; Bedford, Massachusetts)	VEGF and PDGF	Phase I underway
	Durasert Bioerodible TKI (Durasert; EyePoint Pharmaceuticals, Watertown, Massachusetts)	VEGF and PDGF	Pre-clinical investigation
	ENV1305 (Envisia Therapeutics, Morrisville, North Carolina)	VEGF-A	Pre-clinical investigation
	NT-503 (Neurotech, Cumberland, Rhode Island)	VEGF-A	Pre-clinical investigation
Gene therapy	Adeno-associated virus type 2 (AAV2)-sFlt-1 (Genzyme, Cambridge, Massachusetts)	Produces sFlt-1, a soluble isoform of VEGFR-1, and an antagonist of VEGF	Phase I trial completed
	rAAV.SFLT-1 (AVA-101; Avalanche Biotechnologies, Redwood City, California)	Produces sFlt-1, a soluble isoform of VEGFR-1, and an antagonist of VEGF	Phase II trial completed
	ADVM-022 and ADVM-032 (Adverum Biotechnologies, Redwood City, California)	Produces aflibercept-like protein and ranibizumab-like protein	Phase I trial completed
	RGX-314 (REGENXBIO, Rockville, Maryland)	Vector expressing a protein similar to ranibizumab	Phase I/II underway
	Retinostat (Oxford Biomedica, Cowley, Oxford)	Codes for endostatin and angiostatin	Phase I completed
	AAVCAGsCD59 (HMR59; Hemera Biosciences, Waltham, Massachusetts)	Produces CD59	Phase I underway
Oral treatments	AKST4290 (Alkahest, San Carlos, California)	Targets CCR3	Phase II completed
Topical treatments	PAN-90806 (PanOptica, Mount Arlington, New Jersey)	VEGF-A and PDGF	Phase II completed
	Pazopanib (GlaxoSmithKline, Brentford, London)	VEGF-A and PDGF	Phase II completed
	OHR-102 (Squalamine lactate, Ohr Pharmaceutical, New York, New York)	VEGF, PDGF, and b-FGF	Phase II completed
	Regorafenib (Stivarga, Bayer Healthcare, Leverkusen, North-Rhine, Westphalia)	VEGF-A and PDGF	Phase II completed
	LHA510 (Alcon, Geneva, Switzerland)	Tyrosine kinase	Phase II completed

#### Ranibizumab biosimilars

##### Razumab

Razumab (Intas Pharmaceuticals Ltd., Ahmedabad, India), a humanized, monoclonal IgG antibody fragment is the only biosimilar approved in any country for ranibizumab [27]. Razumab was approved in 2015 after displaying efficacy in 103 neovascular AMD patients and is available only in India [27]*.*

##### FYB 201

Currently slated for release in the near future is FYB 201 (Formycon AG, Munich, Germany and Bioeq Gmbh Holzkirchen, Germany) [27]. COLUMBUS-AMD, a phase III randomized control trial in nAMD patients to test FYB 201, met its primary endpoint of non-inferiority in mean change in BCVA at 8 weeks to monthly ranibizumab [28, 29]. Safety was similar to ranibizumab.

##### SB-11

SB-11 (Samsung Bioepsis, Incheon, South Korea) met the primary endpoint of non-inferiority to monthly ranibizumab in a phase III randomized controlled clinical trial in the treatment of neovascular AMD at 4 and 8 weeks [30]. It also showed similar safety.

##### Xlucane

Xlucane (Xbrane Biopharma, Solna, Sweden) is currently under investigation in Xplore, a phase III randomized clinical trial [31].

##### PF582

PF582 (Pfnex, San Diego, California), has been investigated in 25 patients in a phase I/II randomized clinical trial and showed no difference in systemic side effects or visual acuity compared to ranibizumab [32]. A phase 3 trial is planned.

##### CHS3551

CHS3551 (Coherus BioSciences, Redwood City, California), is developing a biosimilar to ranibizumab, which is currently in pre-clinical investigation [33].

#### Aflibercept biosimilars

Unlike ranibizumab, there are no current medications approved as biosimilar alternatives for aflibercept as the patent is in force till at least 2023; however, the following aflibercept biosimilars are under development.

##### FYB203

FYB203 (Formycon AG, Munich, Germany and Bioeq Gmbh Holzkirchen, Germany) is in the pre-clinical stages [34].

##### ALT-L9

ALT-L9 (Alteogen, Deajeon, South Korea) is entering phase I trials [35].

##### MYL1710

MYL1710 (Momenta Pharamaceuticals, Cambridge, MA, and Mylan Pharmacueticals, Canonsburg, PA) is being investigated in phase III trials [36].

##### CHS-2020

CHS-2020 (Coherus BioSciences, Redwood City, California) is in pre-clinical development [37].

#### Bevacizumab biosimilars

##### ONS-5010

ONS-5010 (Lytenava, Outlook Therapeutics, Cranberry Township, New Jersey) has reported a positive phase III trial, the NORSE 1 study (N = 61), to evaluate its efficacy of ONS-5010 dosed monthly as compared to ranibizumab delivered using the PIER study schema of 3 monthly loading doses followed by fixed dosing every 3 months [38]. While there were no statistical differences between the drugs despite the vastly different dosing regimens, 2 of 25 (8%) patients in the ONS-5010 arm achieved > 15 letters best corrected visual acuity (BCVA) at month 11 compared to 5 of 23 (22%) patients in the ranibizumab arm. In the subgroup analysis of treatment-naïve subjects, 2 of 6 (33%) patients on the ONS-5010 arm achieved > 15 letters at month 11 compared to 4 of 13 (31%) patients in the ranibizumab arm. The safety was similar. The confirmatory NORSE 2 study (N = 227) is fully enrolled and underway. Interestingly, this drug would give physcians a FDA approved version of bevacizumab that has been used successfully off-label for years at a higher cost than the fractionated version.

### Pipeline VEGF targets

#### Abicipar pegol

Abicipar pegol (Abicipar; Allergan, Coolock, Dublin) is a mono designed ankyrin repeat protein (monoDARPin) targeting all VEGF-A isoforms [39]. Abicapar injections at fixed 8 or 12 weeks were non-inferior compared to ranibizumab given every 4 weeks in the CEDAR and SEQUOIA studies [39]. However, similar to brolucizumab, abicapar has demonstrated high rates of intraocular inflammation, 15.4%, compared to 0.3% with ranibizumab in the phase 3 studies. Lower rates of inflammation were reported in the bridge MAPLE study, at 8.9%, which was performed after manufacturing changes to reduce host cell impurities were implemented [40]. Despite these promising results and lower drug administration rates, the Food and Drug Administration has delivered a Complete Response Letter for the drug [41]. Additional studies are planned.

#### OPT-302

OPT-302 (Opthea; OPTHEA limited; Victoria, Melbourne) is a VEGF fusion protein with specific domains from human VEGF receptor 1 and 2 combined with the Fc from human IgG1that target VEGF-C and VEGF-D [42]. The usage of OPT-302 in conjunction with aflibercept has shown beneficial improvements as compared to afibercept alone [43]. A phase I trial of 51 patients, 25 of which were treatment naive, evaluated OPT-302 as monotherapy or in combination with ranibizumab [42]. Both medications were administered on a 4-week interval. 54% of the 13 patients on OPT-302 monotherapy did not require anti-VEGF A rescue therapy, and only 38% required one rescue injection [42]. In the phase 2b trial, 366 patients were randomized to a 1:1:1 ratio of either 2 mg (mg) of OPT-302 with 0.5 mg of ranibizumb, 0.5 mg of OPT-302 with 0.5 mg of ranibizumab, or 0.5 mg of ranibizumab alone [44]. The 2 mg of OPT-302 in conjunction with 0.5 mg of ranibizumab showed a significant increase in improvement of best-corrected visual acuity from baseline at 24 weeks as compared to the 0.5 mg of ranibizumab monotherapy. Furthermore, there were higher rates of patients gaining 15 letters or more in the former group, along with less subretinal fluid, intraretinal fluid and choroidal neovascular area [43, 44]. The safety profile between the two groups was comparable [43, 44].

#### Conbercept

Conbercept (Lumitin; Chengdu Kanghong Pharmaceutical Group, Chengdu, Sichuan) is a recombinant human fusion protein of extracellular domains of VEGFR1, VEGFR2 and the portion of Fc IgG1. The difference between aflibercept and conbercept is the addition of VEGF receptor 2 domain 4 which allows the drug to bind VEGF-A, VEGF-B, and PGF tighter than aflibercept. This medication has been evaluated in a phase III, prospective, double-masked, multicenter, and sham-controlled trial in neovascular AMD in China [45]. At 3 months, the conbercept group showed a significant increase in BCVA as compared to sham, with loss of this significant difference at 12 months since patients in the sham group crossed over to receive conbercept after 3 months [45]. The phase 3 trials, PANDA-1 and PANDA-2, are randomized, quadruple-masked, multi-centered trials evaluating three equally distributed arms: 0.5 mg (mg) of conbercept every 8 weeks, 1.0 mg of conbercept every 12 weeks, and 2.0 mg of aflibercept every 8 weeks. The primary endpoint of the study is mean change in BCVA at 36 weeks [46]. The studies are fully enrolled with results expected in 2021.

#### KSI-301

KSI-301 (Kodiak Sciences, Palo Alto, CA) is anti-VEGF antibody biopolymer conjugate, composed of two parts—a humanized anti-VEGF antibody with CDR similar to ranibizumab and a phosphorylcholine-based polymer. The latter portion of the molecule increases intraocular stability and durability in the eye [4, 7]. A phase Ib, randomized, open-label study with KSI-301 (utilizing both 2.5 mg and 5.0 mg) evaluated patients with neovascular AMD, diabetic macular edema, and retinal vein occlusion. In particular, 55% of the patients enrolled in the neovascular AMD arm have gone 6 months without a re-treatment [47, 48]. DAZZLE, a phase III, randomized, double-blinded, noninferiority multicenter trial will investigate KSI-301 in 368 patients with neovascular AMD [49]. This study will evaluate 5 mg dosage injections of KS1-301 at 12, 16, or 20-week intervals after an initial three loading doses in comparison to patients receiving aflibercept at 8-week intervals after three initial monthly loading doses. After 1 year of treatment, patients in the aflibercept arm will be randomized to either KSI-301 or aflibercept and reviewed at 96 weeks. The primary outcome of this trial will assess the change in BCVA from baseline at 52 weeks.

### Pipeline combination VEGF and anti- platelet derived growth factor (PDGF) therapies

In addition to the previously described pathways in neovascular AMD, pericytes may have a role in limiting the effects of anti-VEGF therapy. Therefore, there has been a concerted effort in producing medications which target both the anti-VEGF and anti-PDGF pathways to enhance medication response [4, 7].

#### CLS-AX

CLS-AX (Ataxinib; Clearside Biomedical, Alpharetta, Georgia) is currently investigating axitinib, a tyrosine kinase inhibitor, injected into the suprachoroidal space. This medication targets both VEGF and PDGF which are tyrosine kinase receptors [50].

#### Pegpleranib

Pegpleranib (Fovista; Ophthotech, Princeton, New Jersey) is a 32-mer-pegylated DNA aptamer. This structure binds to both PDGF-BB and PDGF-AB in order to block their binding to PDGF tyrosine kinase receptors on pericytes [4, 7, 51]. Initially, a phase IIb trial showed promising results in patients treated with a combination of 1.5 mf of pegpleranib and 0.5 mg of ranibizumab versus monotherapy with ranibizumab [52]. However, these results were not reproduced in the phase III trials; therefore, development of the drug was halted [53].

#### Rinucumab

Rinucumab (Regeneron, Tarrytown, New York) is an anti-PDGF receptor-β antibody, designed for co-injection with aflibercept and studied in the phase II CAPELLA trial. This trial failed to meet its primary endpoint of improved BCVA at 12 weeks [53].

#### DE-120

DE-120 (Santen Pharmaceuticals, Kita-Ku, Osaka) is a dual tyrosine kinase inhibitor of VEGF-A and PDGF. However, Santen has discontinued this drug development after poor study results [54].

#### X-82

X-82 (Tyrogenex, Rockville, Maryland) is an oral PDGF and VEGF-A inhibitor. It has been studied in a phase I trial in 35 patients; of these, 10 did not complete the trial due to systemic side effects, which reversed with drug termination. Of the 25 who did complete the trial, 24 had improvement or maintenance of their vision, and 60% of these did not require a rescue intravitreal injection of ranibizumab [55]. APEX, a phase II study, has evaluated various doses of X-82 in conjunction with pro re nata (PRN) anti-VEGF injections in 157 patients against placebo [56]. The study showed non-inferiority of X-82, but given the side effect profile of the medication, it was not recommended for the treatment of neovascular AMD [56].

### Pipeline combination VEGF and tissue factor therapies

Additionally, tissue factor is unregulated in the production of choroidal neovascular membranes and linked to neovascular AMD. Therefore, targeting this may improve anatomic and visual outcomes [7].

#### ICON-1

ICON-1 (Iconic Therapeutics, San Francisco, California) is a combination product of factor VIIIa linked with the Fc portion of human IgG1. This molecule acts in two ways. In addition to binding to tissue factor, it also binds to Natural Killer cells which attack CNVs. Additionally, this therapy can be used in combination with other anti-VEGF medications. ICON-1 has been evaluated in a phase I, open-label trial in 18 patients with neovascular AMD, and met the primary endpoints of safety [57]. Further investigation of ICON-1 have been undertaken with EMERGE, a phase II, randomized, double-masked study in 88 patients with CNV in neovascular AMD. These patients were randomized to a 1:1:1 ratio of either 0.5 mg ranibizumab, 0.3 mg of ICON-1, or a combination of the two. The combination arm showed the greatest decrease in CNV, while BCVA was the same between the combination arm and the ranibizumab arm [58]. DECO is a phase II randomized, open-label, parallel study in 15 patients with neovascular AMD treated with aflibercept initially and then switched to maintenance therapy, either with 0.6 mg of ICON-1 or 2 mg of aflibercept. The primary endpoint of this study will assess the change in CNV area from baseline to 9 months [59].

### Pipeline combination VEGF and Tie-2 receptor therapies

The Tie-2 tyrosine kinase receptor is expressed on endothelial cells, and activating this receptor allows for decreased vascular permeability, inflammation, and angiogenesis. Angiopoietin-1 (Ang-1) binds to the Tie-2 receptor in order to decrease vascular leakage, while angiopooetin-2 (Ang-2) is a competitive antagonist to Ang-1, thus increasing leakage, inflammation, and angiogenesis. Multiple drugs activating various portions of this pathway are in development [4, 7, 60].

#### Faricimab

Faricimab (Roche/Genentech, San Francisco, CA) is a bispecific antibody which targets both VEGF-A and Ang-2 [4, 7]. AVENUE is phase II, randomized, double-masked trial in 237 patients comparing various dosages of faricimab compared to ranibizumab, as well as compared to the combination of both drugs. The 1.5 mg of faricimab every 4 weeks showed the best gain in visual acuity, while the combination arm showed the greatest reduction in central subfield thickness [61]. STAIRWAY is also a phase II, randomized, double masked trial comparing faricimab to ranibizumab in neovascular AMD, and showed visual acuity was maintained in those treated with faricimab [62]. Currently, LUCERENE and TENAYA are two phase III, randomized, triple masked trials evaluating 640 patients with neovascular AMD treated with faricimab or aflibercept. The primary outcome was met with faricimab up to every 16 weeks was non-inferior to aflibercept dosed every 8 weeks [63, 64]. Almost 45% of patients in both studies were treated at 16-week intervals during the first 48 weeks. The treatment was well tolerated.

#### REGN910

REGN910 (Nevascumab; Regeneron, Tarrytown, New York) inhibits Ang-2 and was used in conjunction with aflibercept. Promising results were obtained in phase I trials [65]. ONYX, a phase II study, compared the combination of these two drugs to aflibercept monotherapy, but the trial failed to meet its primary endpoint of a significant improvement in BCVA change at 36 weeks [66]. Development has been halted.

#### ARP-1536

ARP-1536 (Aerpio Therapeutics, Blue Ash, Ohio) inhibits vascular endothelial protein tyrosine phosphatase (VE-PTP), which itself inhibits Tie-2. Therefore, ARP-1536 causes increased activity of Tie-2 and decreased vascular permeability. It has shown efficacy in pre-clinical trials [67].

#### Endoglin

DE-122 (Carotuximab; Santen, Kita-Ku, Osaka, and TRACON Pharmaceuticals, San Diego, CA) is an anti-endoglin antibody. Like PDGF, endoglin is expressed by endothelial cells and is expressed by choroidal neovascular membranes [43]. PAVE, a phase I/II trial in 12 patients tested four dosages of DE-122: 0.5 mg, 1.0 mg, 2.0 mg or 4.0 mg, with improvement in the three higher dosages at 90 days [68]. A phase II trial, AVANTE, was a randomized, double-masked control study comparing intravitreal DE-122 in combination with ranibizumab versus ranibizumab alone in 76 patients. However, the trial showed no significant difference in the combination therapy and manufacturing of the medication was stopped [69].

#### AXT-107

AXT-107 (AsclepiX Therapeutics, Jersey City, New Jersey), inhibits both VEGFR2 and other growth factor signaling via receptor tyrosine kinase interactions, and increasing Tie2 activity. The drug has been studied in preclinical cohorts and been shown to decrease blood vessel leakage in neovascular AMD [70] It is currently in clinical testing.

### Sustained treatments

#### Ranibizumab port delivery system (PDS)

In addition to serial treatments, another exciting opportunity for treatment includes sustained drug deliver systems. Ranibizumab PDS (Genentech, San Francisco, CA) is a permanent, surgically implanted intraocular device [71]. This device allows for clinic-based refills instead of another surgical procedure. This PDS system has shown promising results in the trials LADDER and ARCHWAY [71, 72]. LADDER, a phase II trial, showed comparable visual outcomes in patients treated with the 100 mg/ml treatment arm and the monthly injection arm [71]. While no ocular adverse events were noted in the monthly group, 16 of 179 implant eyes experienced ocular adverse events, the most common of which were vitreous hemorrhage, occurring in 7 (3.9%) of eyes [71]. Endophthalmitis occurred in 3 (1.7%) of eyes [71]. ARCHWAY, a phase III trial, utilized the 100 mg/ml surgical implant and showed non-inferior outcomes and equivalent visual acuity compared to monthly ranibizumab injections [73]. Additionally, 98% of patients were able to last 6 months without refills on medication [73]. In this study, the most common adverse event was a conjunctival bleb, occurring in 6.5% of patients, while vitreous hemorrhage occurred in 5.2%, all of which resolved without intervention. Four cases (1.4%) developed endophthalmitis, three of which returned to baseline vision [74].

#### GB-102

GB-102 (Sunitinib maleate; GrayBug Vision, Redwood City, CA) is a tyrosine kinase inhibitor, which works against VEGF-A and PDGF. The tyrosine kinase inhibitor is contained in a biodegradable PLGA polymer which allows for sustained delivery, only requiring re-treatment every 6 months. ADAGIO, a phase I/IIa was an open-label, single dose trial in 32 patients of either 0.25, 0.5, 1 or 2 mg of GB-102. This trial showed safety and 68% of patients requiring no rescue treatment for 6 months. However, the 2 mg dosage group had microparticle migration into the anterior chamber, which has prompted a newer manufacturing response to eliminate this phenomenon [75]. ALTISSIMO, a phase IIb, randomized, single masked trial, comparing 1 mg or 2 mg of GB-102 to 2 mg of aflibercept is underway currently.

#### OTX-TKI

OTX-TKI, a hydrogel depot of the tyrosine kinase inhibitor, sunitinib (Ocular Therapeutix; Bedford, Massachusetts) is currently in early clinical testing which has a proposed efficacy window of 6 months. The hydrogel maintains therapeutic levels through a hydrogel network through which the tyrosine kinase inhibitor can slowly diffuse. This medication is currently in phase I trials with no serious side adverse effects noted [76].

#### Durasert Bioerodible TKI

Durasert Bioerodible TKI (Durasert; EyePoint Pharmaceuticals, Watertown, Massachusetts) utilizes an implant system to release tyrosine kinase inhibitors of both VEGF and PDGF. This is currently in preclinical stages [4, 7, 77].

#### AR13503/ENV1305

ENV1305 (Envisia Therapeutics, Morrisville, North Carolina) is a long-term, sustained release agent of anti-VEGF-A. The release of this product is dependent on its nanoparticle technology, which has been bought by Arie Pharmaceuticals, (Durham, North Carolina) to create AR13503 [78]. This drug is currently in the pre-clinical development stages.

#### NT-503

NT-503 (Neurotech, Cumberland, Rhode Island) functions through an intravitreal implant through which an RPE cell line produces a VEGF-A fusion protein. However, production has halted due to insufficient efficacy and increased rescue treatment requirements [79].

#### Gene therapy

Gene therapy provides a promising treatment alternative for neovascular AMD for multiple reasons, including a tight blood-ocular barrier and immune privileged state [4, 7]. Gene therapy offers a more permanent solution even compared to sustained release delivery systems, as the capsid enable the translation of viral genetic material into constantly expressed proteins to which can inhibit and modify the pathogenesis of neovascular AMD.

#### AAV2-sFLT01

Adeno-associated virus type 2 (AAV2)-sFlt-1 (Genzyme, Cambridge, Massachusetts) combines a viral vector with a plasmid, which produces sFlt-1, a soluble isoform of VEGFR-1, and an antagonist of VEGF [80, 81]. Phase I trial data in 19 patients shows tolerability but a varying degree of expression, therefore, the drug has not entered further developmental stages [82].

#### AVA-101

rAAV.SFLT-1 (AVA-101; Avalanche Biotechnologies, Redwood City, California), utilizes the same concept of AAV2-sFLTO1, but instead uses a subretinal approaches opposed to an intravitreal injection. Phase I results were initially promising [83, 84]. However, the results were less promising in the phase IIb trial with no difference in visual acuity between the control group and the intervention group [85].

#### ADVM-022/ADVM-032

ADVM-022 and ADVM-032 (Adverum Biotechnologies, Redwood City, California) utilize a modified AAV2 vector specialized for intravitreal injections, with ADVM-022 producing an aflibercept-like protein and ADVM-032 producing a ranibizumab-like protein [80]. These have shown inhibition of laser induced choroidal neovascularization in primate models [80, 86]. ADVM-022 has been shown to be safe and efficacious in a phase I trial, OPTIC, with many patients not requiring any rescue anti-VEGF injections [87].

#### RGX-314

RGX-314 (REGENXBIO, Rockville, Maryland), utilizes adeno associated virus serotype 8 (AAV8) as its vector expressing a protein similar to ranibizumab [88]. Currently, this gene therapy is under investigation in phase I/IIa open-label dose escalation studies with vitrectomy and subretinal injection and another study evaluating suprachroidal injection. There are five cohorts of doses included in the subretinal surgery study, and the primary endpoint is safety at 26 weeks. Initial results have shown a dose-dependent relationship with protein expression and anti-VEGF rescue injections. Promising results have been shown in cohort three, four and five, with cohort three having 50% of patients remaining free of anti-VEGF injections at 18 months [89].

#### Retinostat

Retinostat (Oxford Biomedica, Cowley, Oxford) utilizes a recombinant equine infectious anemia virus (EIAV), which codes for two proteins-endostatin and angiostatin through a subretinal injection [90]. GEM, the phase 1 trial, evaluated 21 patients with neovascular AMD with this therapy and found no adverse effects [91].

#### AAVCAGsCD59

AAVCAGsCD59 (HMR59; Hemera Biosciences, Waltham, Massachusetts) is an intravitreal injection of AAV2 producing a protein for soluble CD59, which blocks formation of the membrane attack complex (MAC), the last stage of the complement pathway. This has been tested in mice and prevented laser provoked choroidal neovascular membranes from forming [92]. The gene therapy is now being evaluated in a phase I, open-label study in conjunction with anti-VEGF injections and has demonstrated a reduced treatment burden with anti-VEGF [93].

#### Oral treatments

##### AKST4290

AKST4290 (Alkahest, San Carlos, California) is an oral treatment targeted against CCR3, the receptor for eotaxin, which is also expressed on endothelial cells. Eotaxin is linked to membrane permeability and the pathogenesis of neovascular AMD [43]. Two phase IIa trials have evaluated this medication in both treatment naïve and treatment resistant neovascular AMD patients [94]. 72–83% of patients had stabilization or improvement of BCVA with treatment [94].

#### Topical treatments

##### PAN-90806

PAN-90806 (PanOptica, Mount Arlington, New Jersey) is a topical medication, which acts as a tyrosine kinase inhibitor of VEGF-A and PDGF. Phase I/II randomized, double masked, uncontrolled studies have reached primary endpoint measures of safety. Half of the patients did not require rescue therapy, with over 80% showing stabilization or improvement [95]. This medication may provide a possible monotherapy agent for neovascular AMD in select patients in the future.

##### Pazopanib

Pazopanib (GlaxoSmithKline, Brentford, London) is a topical formulation of a tyrosine kinase inhibitor, which inhibits both VEGF-A and PDGF. The drop has been evaluated in a few trials for its efficacy in treating wet AMD. In a multicentre, double-masked trial 70 patients with minimally classic or occult subfoveal choroidal neovascularization were assigned to three groups: 5 mg/ml daily, 2 mg/ml three times a day, or 5 mg/ml three times a day for a treatment duration of 28 days. Overall, there was no significant decrease from baseline central retinal thickness when the group was analyzed overall, but a sub-cohort of patients, those with CFH-TT genotype had a significant decrease with the 5 mg/ml three times a day treatments [96].

The drop has also been evaluated in a phase IIb trial, evaluating 510 subjects, which showed that in combination with ranibizumab it was shown to be non-inferior, however the treatment burden with ranibizumab did not decrease by the pre-determined 50%, and there were no significant differences between the groups in terms of choroidal neovascular membrane size or characteristics of imaging, or in baseline retinal thickness or morphology. In this analysis, the complement factor H genotype did not have an impact on the type of response from pazopanib or ranibizumab. Further development has been halted [97].

##### OHR-102

OHR-102 (Squalamine lactate, Ohr Pharmaceutical, New York, New York) is a topical eye drop that interferes with the signaling of multiple angiogenic factors receptors, including VEGF, PDGF, and b-FGF. IMPACT, a phase II study compared the combination therapy of squalamine lactate and ranibizumab with ranibizumab monotherapy, however it failed to meet its primary endpoint of decreased treatment burden of ranibizumab. Interestingly, a small subset of patients, with occult CNV, there was a BCVA increase with the combination group [98]. Further development has been halted.

##### Regorafenib

Regorafenib (Stivarga, Bayer Healthcare, Leverkusen, North-Rhine, Westphalia) is a topical therapy, which inhibits multiple kinases in order to impact the VEGF-A and PDGF receptors. DREAM, the phase IIa study, failed to show efficacy [99]. Further development has been halted.

##### LHA510

LHA510 (Alcon, Geneva, Switzerland) is a topical tyrosine kinase inhibitor that was evaluated as maintenance therapy with ranibizumab in a phase 2 study [100]. The results did not demonstrate efficacy and further development has been halted.

## Conclusion

Neovascular age related macular degeneration can have devastating visual, social and financial impacts. It has a complex pathogenesis and has various treatment options. Traditionally, modalities have aimed at the anti-VEGF pathway and yielded better results than prior laser, sham and surgical interventions. However, the management burden, financial implications, and patient compliance factors may limit the more regimented treatment options. Current therapeutic avenues are investigating additional cytokine pathways, including PDGF and Tie-2. Also, energies are being directed into longer lasting depots and gene therapy, which can limit the frequency of visits and ease the social burdens surrounding the disease.

## Data Availability

Data sharing is not applicable to this article as no datasets were generated or analysed during the current study.

## References

[1] Friedman DS, O'Colmain BJ, Muñoz B (2004). Prevalence of age-related macular degeneration in the United States. Arch Ophthalmol.

[2] Wong WL, Su X, Li X (2014). Global prevalence of age-related macular degeneration and disease burden projection for 2020 and 2040: a systematic review and meta-analysis. Lancet Glob Health.

[3] Ferris FL, Fine SL, Hyman L (1984). Age-related macular degeneration and blindness due to neovascular maculopathy. Arch Ophthalmol.

[4] Al-Khersan H, Hussain RM, Ciulla TA, Dugel PU (2019). Innovative therapies for neovascular age-related macular degeneration. Expert Opin Pharmacother.

[5] Gass JD (1967). Pathogenesis of disciform detachment of the neuroepithelium. Am J Ophthalmol.

[6] Leung DW, Cachianes G, Kuang WJ, Goeddel DV, Ferrara N (1989). Vascular endothelial growth factor is a secreted angiogenic mitogen. Science.

[7] Hussain RM, Ciulla TA (2017). Emerging vascular endothelial growth factor antagonists to treat neovascular age-related macular degeneration. Expert Opin Emerg Drugs.

[8] Rosenfeld PJ, Brown DM, Heier JS (2006). Ranibizumab for neovascular age-related macular degeneration. N Engl J Med.

[9] Heier JS, Brown DM, Chong V (2012). Intravitreal aflibercept (VEGF trap-eye) in wet age-related macular degeneration. Ophthalmology.

[10] Arevalo JF, Lasave AF, Wu L (2016). Intravitreal bevacizumab for choroidal neovascularization in age related macular degeneration: 5 year results of the pan American collaborative retina study group. Retina.

[11] Mones J, Singh RP, Bandello F, Souied E, Liu X, Gale R (2020). Undertreatment of neovascular age-related macular degeneration after 10 years of anti-vascular endothelial growth factor therapy in the real world: the need for a change of mindset. Ophthalmologica.

[12] Cohen SY, Mimoun G, Oubraham H (2013). Changes in visual acuity in patients with wet age-related macular degeneration treated with intravitreal ranibizumab in daily clinical practice: the LUMIERE study. Retina.

[13] Holz FG, Tadayoni R, Beatty S (2015). Multi-country real-life experience of anti-vascular endothelial growth factor therapy for wet age-related macular degeneration. Br J Ophthalmol.

[14] Smith AG, Kaiser PK (2014). Emerging treatments for wet age-related macular degeneration. Expert Opin Emerg Drugs.

[15] Doggrell SA (2005). Pegaptanib: the first antiangiogenic agent approved for neovascular macular degeneration. Expert Opin Pharmacother.

[16] Holash J, Davis S, Papadopoulos N (2002). VEGF-Trap: a VEGF blocker with potent antitumor effects. Proc Natl Acad Sci USA.

[17] Martin DF, Maguire MG, Fine SL (2012). Ranibizumab and bevacizumab for treatment of neovascular age-related macular degeneration: two-year results. Ophthalmology.

[18] Ferrara N, Damico L, Shams N, Lowman H, Kim R (2006). Development of ranibizumab, an anti-vascular endothelial growth factor antigen binding fragment, as therapy for neovascular age-related macular degeneration. Retina.

[19] Brown DM, Kaiser PK, Michels M (2006). Ranibizumab versus verteporfin for neovascular age-related macular degeneration. N Engl J Med.

[20] Ohr M, Kaiser PK (2012). Aflibercept in wet age-related macular degeneration: a perspective review. Ther Adv Chronic Dis.

[21] Khanna S, Komati R, Eichenbaum DA, Hariprasad I, Ciulla TA, Hariprasad SM (2019). Current and upcoming anti-VEGF therapies and dosing strategies for the treatment of neovascular AMD: a comparative review. BMJ Open Ophthalmol.

[22] Dugel PU, Jaffe GJ, Sallstig P (2017). Brolucizumab versus aflibercept in participants with neovascular age-related macular degeneration: a randomized trial. Ophthalmology.

[23] Dugel PU, Koh A, Ogura Y (2020). HAWK and HARRIER: phase 3, multicenter, randomized, double-masked trials of brolucizumab for neovascular age-related macular degeneration. Ophthalmology.

[24] Baumal CR, Spaide RF, Vajzovic L (2020). Retinal vasculitis and intraocular inflammation after intravitreal injection of brolucizumab. Ophthalmology.

[25] Sharma A, Kumar N, Bandello F, Loewenstein A, Kuppermann BD (2019). Need of education on biosimilars amongst ophthalmologists: combating the nocebo effect. Eye.

[26] Sharma A, Kumar N, Kuppermann BD, Bandello F, Loewenstein A (2020). Understanding biosimilars and its regulatory aspects across the globe: an ophthalmology perspective. Br J Ophthalmol.

[27] Sharma A, Reddy P, Kuppermann BD, Bandello F, Lowenstein A (2018). Biosimilars in ophthalmology: “Is there a big change on the horizon?”. Clin Ophthalmol.

[28] FY18 results: FYB201 launch on track for 2021. 2019. https://www.edisongroup.com/publication/fy18-results-fyb201-launch-on-track-for-2021/24222/. Accessed 1 Jan 2020.

[29] Efficacy and safety of the biosimilar ranibizumab FYB201 in comparison to lucentis in patients with neovascular age-related macular degeneration (COLUMBUS-AMD), ClinicalTrials.gov Identifier: NCT02611778. 2020. https://clinicaltrials.gov/ct2/show/NCT02611778. Accessed 1 Jan 2020.

[30] A study to compare SB11 (proposed ranibizumab biosimilar) to lucentis in subjects with neovascular age-related macular degeneration (AMD). ClinicalTrials.gov Identifier: NCT03150589. https://clinicaltrials.gov/ct2/show/NCT03150589. Accessed 1 Jan 2020.

[31] Comparing the efficacy and safety of biosimilar candidate xlucane versus lucentis® in patients with nAMD (XPLORE). ClinicalTrials.gov Identifier: NCT03805100. https://clinicaltrials.gov/ct2/show/NCT03805100. Accessed 1 Jan 2020.

[32] Pfenex to regain full rights to PF582 and announces phase 1/2 results 2019. https://www.prnewswire.com/news-releases/pfenex-to-regain-full-rights-to-pf582-and-announces-phase-12-results-300310159.html. Accessed 1 Jan 2020.

[33] https://www.coherus.com/products-and-pipeline/. Accessed 1 Jan 2020.

[34] FYB203. 2018. https://www.bioeq.com/development-programs/fyb203/. Accessed 1 Jan 2020.

[35] Alteogen Inc. Gets IND approval for a clinical study in Korea for Eylea biosimilar (ALT-L9). 2019. https://www.biosimilardevelopment.com/doc/alteogen-inc-gets-ind-approval-for-a-clinical-study-in-korea-for-eylea-biosimilar-alt-l-0001. Accessed 1 Jan 2020.

[36] Comparative study to evaluate the efficacy and safety of MYL-1701P and Eylea® in subjects with diabetic macular edema, ClinicalTrials.gov Identifier: NCT03610646. 2018. https://clinicaltrials.gov/ct2/show/NCT03610646. Accessed 1 Jan 2020.

[37] Addressing important unmet needs. 2020. https://www.coherus.com/products-and-pipeline/. Accessed 1 Jan 2020.

[38] Clinical policy: bevacizumab (Avastin, Mvasi, Zirabev). 2014. https://pharmacy.envolvehealth.com/content/dam/centene/envolve-pharmacy-solutions/pdfs/PriorAuthorizationGuidelines/pa-guidelines-september2019/PAGuidelinesQ420192/ERX.SPA.86Bevacizumab(Avastin,Mvasi,Zirabev)12.01.19.pdf. Accessed 1 Jan 2020.

[39] Kunimoto D, Yoon YH, Wykoff CC (2020). Efficacy and safety of abicipar in neovascular age-related macular degeneration: 52-week results of phase 3 randomized controlled study. Ophthalmology.

[40] Hussain RM, Weng CY, Wykoff CC, Gandhi RA, Hariprasad SM (2020). Abicipar pegol for neovascular age-related macular degeneration. Expert Opin Biol Ther.

[41] Abicipar pegol not approved for treatment of wet AMD. 2020. https://www.healio.com/news/ophthalmology/20200626/abicipar-pegol-not-approved-for-treatment-of-wet-amd. Accessed 20 July 2020.

[42] Dugel PU, Boyer DS, Antoszyk AN (2020). Phase 1 study of OPT-302 inhibition of vascular endothelial growth factors C and D for neovascular age-related macular degeneration. Ophthalmol Retina.

[43] Samanta A, Aziz AA, Jhingan M, Singh SR, Khanani A, Chhablani J (2020). Emerging therapies in neovascular age-related macular degeneration in 2020. Asia Pac J Ophthalmol.

[44] A dose-ranging study of intravitreal OPT-302 in combination with ranibizumab, compared with ranibizumab alone, in participants with neovascular age-related macular degeneration (wet AMD). 2017.

[45] Liu K, Song Y, Xu G (2019). Conbercept for treatment of neovascular age-related macular degeneration: results of the randomized phase 3 PHOENIX study. Am J Ophthalmol.

[46] Efficacy and safety trial of conbercept intravitreal injection for neovascular age-related macular degeneration (PANDA-1). 2020. https://clinicaltrials.gov/ct2/show/NCT03577899. Accessed 20 July 2020.

[47] Do D. Update on clinical studies of KSI-301: a novel anti-VEGF antibody biopolymer conjugate with potential for extended durability in wet AMD. In: Angiogenesis, exudation and degeneration. 2020.

[48] Exploratory study to investigate the bioactivity, ocular and systemic safety, tolerability, and pharmacokinetics following single and multiple intravitreal administrations of KSI-301 in subjects with wAMD, DME and RVO. https://clinicaltrials.gov/ct2/show/NCT03790852?term=ksi-301&draw=2&rank=1. Accessed 20 June 2020.

[49] A study to evaluate the efficacy and safety of KSI-301, an anti-VEGF antibody biopolymer conjugate, versus aflibercept in patients with neovascular (wet) age-related macular degeneration. (DAZZLE). 2019. https://clinicaltrials.gov/ct2/show/study/NCT04049266. Accessed 20 June 2020.

[50] Clearside Biomedical, Inc. Redirects pre-clinical AMD research resources toward ongoing DME clinical development program. 2017. https://ir.clearsidebio.com/news-releases/news-release-details/clearside-biomedical-inc-redirects-pre-clinical-amd-research.

[51] Drolet DW, Green LS, Gold L, Janjic N (2016). Fit for the eye: aptamers in ocular disorders. Nucleic Acid Ther.

[52] Jaffe GJ, Ciulla TA, Ciardella AP (2017). Dual antagonism of PDGF and VEGF in neovascular age-related macular degeneration: a phase IIb, multicentre, randomized controlled trial. Ophthalmology.

[53] Dunn EN, Hariprasad SM, Sheth VS (2017). An overview of the fovista and rinucumab trials and the fate of anti-PDGF medications. Ophthalmic Surg Lasers Imaging Retina.

[54] Jaffe GJ. Combination therapy for neovascular AMD retina today. 2016.

[55] Jackson TL, Boyer D, Brown DM (2017). Oral tyrosine kinase inhibitor for neovascular age-related macular degeneration: a phase 1 dose-escalation study. JAMA Ophthalmol.

[56] Cohen MN, O'Shaughnessy D, Fisher K (2020). APEX: a phase II randomised clinical trial evaluating the safety and preliminary efficacy of oral X-82 to treat exudative age-related macular degeneration. Br J Ophthalmol.

[57] Wells JA, Gonzales CR, Berger BB, Gonzalez VH, Sippy BD, Burian G (2018). A phase 1, open-label, dose-escalation trial to investigate safety and tolerability of single intravitreous injections of ICON-1 targeting tissue factor in wet AMD. Ophthalmic Surg Lasers Imaging Retina.

[58] Gonzales CR, Burian G (2017). A phase 2 study (EMERGE) evaluating repeated intravitreal administration of ICON-1 in patients with choroidal neovascularization (CNV) secondary to age-related macular degeneration (AMD). Investig Ophthalmol Vis Sci.

[59] Open-label study of intravitreal ICON-1 in patients with choroidal neovascularization secondary to age-related macular degeneration (AMD)—full text view. 2020. https://clinicaltrials.gov/ct2/show/NCT03452527. Accessed 20 June 2020.

[60] Hansen TM, Singh H, Tahir TA, Brindle NP (2010). Effects of angiopoietins-1 and -2 on the receptor tyrosine kinase Tie2 are differentially regulated at the endothelial cell surface. Cell Signal.

[61] A proof-of-concept study of faricimab (RO6867461) in participants with choroidal neovascularization (CNV) secondary to age-related macular degeneration (AMD) (AVENUE). https://clinicaltrials.gov/ct2/show/NCT03823300. Accessed 20 June 2020.

[62] Study to evaluate faricimab (RO6867461; RG7716) for extended durability in the treatment of neovascular age related macular degeneration (nAMD). https://clinicaltrials.gov/ct2/show/NCT03038880. Accessed 20 June 2020.

[63] A study to evaluate the efficacy and safety of faricimab in participants with neovascular age-related macular degeneration (TENAYA). https://clinicaltrials.gov/ct2/show/NCT03823287. Accessed 20 June 2020.

[64] A study to evaluate the efficacy and safety of faricimab in participants with neovascular age-related macular degeneration (LUCERNE). https://clinicaltrials.gov/ct2/show/NCT03823300. Accessed 20 June 2002.

[65] Boyer D. Aflibercept combination therapies: rinucumab/aflibercept and nesvacumab/aflibercept. In: American academy of ophthalmology annual meeting; 2016.

[66] Anti-angiopoeitin 2 plus anti-vascular endothelial growth factor as a therapy for neovascular age related macular degeneration: evaluation of a fixed combination intravitreal injection (ONYX). https://clinicaltrials.gov/ct2/show/NCT02713204. Accessed 20 June 2020.

[67] ARP-1536 retinopathy/nephropathy. https://aerpio.com/pipeline/arp-1536-diabetic-retinopathy-nephropathy/. Accessed 20 June 2020.

[68] Inc S. Santen presents phase I/II data on DE-122 (carotuximab) in patients with refractory wet age-related macular degeneration. 2018. https://www.prnewswire.com/news-releases/santen-presents-phase-iii-data-on-de-122-carotuximab-in-patients-with-refractory-wet-age-related-macular-degeneration-300597011.html.

[69] Santen and TRACON discontinue development of DE-122 for wet age-related macular degeneration. 2020. https://www.biospace.com/article/releases/santen-and-tracon-discontinue-development-of-de-122-for-wet-age-related-macular-degeneration/.

[70] AsclepiX Therapeutics. Our pipeline. 2020. https://asclepix.com/pipeline/.

[71] Campochiaro PA, Marcus DM, Awh CC (2019). The port delivery system with ranibizumab for neovascular age-related macular degeneration: results from the randomized phase 2 ladder clinical trial. Ophthalmology.

[72] Campochiaro PA, Brown DM, Awh CC (2011). Sustained benefits from ranibizumab for macular edema following central retinal vein occlusion: twelve-month outcomes of a phase III study. Ophthalmology.

[73] Phase III data show port delivery system with ranibizumab enabled over 98% of patients to go six months between treatments for neovascular age-related macular degeneration. 2020. https://www.biospace.com/article/releases/phase-iii-data-show-port-delivery-system-with-ranibizumab-enabled-over-98-percent-of-patients-to-go-six-months-between-treatments-for-neovascular-age-related-macular-degeneration/.

[74] ASRS 2020: PDS with ranibizumab signals paradigm shift in treatment of neovascular AMD. 2020. https://www.modernretina.com/view/asrs-2020-pds-with-ranibizumab-signals-paradigm-shift-in-treatment-of-neovascular-amd.

[75] GB-102 for treatment of nAMD. http://www.graybug.com/pipeline-technology/gb-102/. Accessed 20 June 2020.

[76] Bisht R, Nirmal S, Agrawal R, Jain GK, Nirmal J (2020). Injectable in-situ gel depot system for targeted delivery of biologics to the retina. J Drug Target.

[77] Product pipeline. https://eyepointpharma.com/pipeline/. Accessed 20 June 2020.

[78] AR-13503 implant. http://ec2-18-188-253-102.us-east-2.compute.amazonaws.com/rd/pipeline/ar-13503/. Accessed 6 July 2020.

[79] NT-503 ECT. http://www.neurotechusa.com/nc-503-ect.html. Accessed 20 June 2020.

[80] In pursuit of gene therapy for AMD an update on investigators’ efforts to achieve effective sustained-release treatments. 2020. https://www.ophthalmologymanagement.com/issues/2017/april-2017/in-pursuit-of-gene-therapy-for-amd.

[81] Pechan P, Rubin H, Lukason M (2009). Novel anti-VEGF chimeric molecules delivered by AAV vectors for inhibition of retinal neovascularization. Gene Ther.

[82] Heier JS, Kherani S, Desai S (2017). Intravitreous injection of AAV2-sFLT01 in patients with advanced neovascular age-related macular degeneration: a phase 1, open-label trial. Lancet.

[83] Constable IJ, Lai CM, Magno AL (2017). Gene therapy in neovascular age-related macular degeneration: three-year follow-up of a phase 1 randomized dose escalation trial. Am J Ophthalmol.

[84] Rakoczy EP, Lai CM, Magno AL (2015). Gene therapy with recombinant adeno-associated vectors for neovascular age-related macular degeneration: 1 year follow-up of a phase 1 randomised clinical trial. Lancet.

[85] Constable IJ, Pierce CM, Lai CM (2016). Phase 2a randomized clinical trial: safety and post hoc analysis of subretinal rAAV.sFLT-1 for wet age-related macular degeneration. EBioMedicine.

[86] Blumenkranz MS. A single intravitreal administration of either ADVM-022 or ADVM-032 demonstrated comparable efficacy to conventional intravitreal anti-VEGF therapy for inducing regression of CNV lesions. In: The retina society 2016 annual meeting. 2016.

[87] Adverum biotechnologies provides update on OPTIC phase 1 trial for ADVM-022 in wet AMD. http://investors.adverum.com/news-releases/news-release-details/adverum-biotechnologies-provides-update-optic-phase-1-trial-advm. Accessed 6 July 2020.

[88] Siddiqui FAAK, A. Gene therapy for neovascular AMD: an update on ongoing clinical trials. Retinal Physician; 2020.

[89] REGENXBIO Reports second quarter 2019 financial and operating results and additional positive interim phase I/IIa trial update for RGX-314 for the treatment of wet AMD. Press release. 2019. https://www.prnewswire.com/news-releases/regenxbio-reports-second-quarter-2019-financial-and-operating-results-and-additional-positive-interim-phase-iiia-trial-update-for-rgx-314-for-the-treatment-of-wet-amd-300898256.html.

[90] Binley K, Widdowson PS, Kelleher M (2012). Safety and biodistribution of an equine infectious anemia virus-based gene therapy, RetinoStat(®), for age-related macular degeneration. Hum Gene Ther.

[91] Campochiaro PA, Lauer AK, Sohn EH (2017). Lentiviral vector gene transfer of endostatin/angiostatin for macular degeneration (GEM) study. Hum Gene Ther.

[92] Cashman SM, Ramo K, Kumar-Singh R (2011). A non membrane-targeted human soluble CD59 attenuates choroidal neovascularization in a model of age related macular degeneration. PLoS ONE.

[93] AAVCAGsCD59 for the treatment of Wet AMD. https://clinicaltrials.gov/ct2/show/NCT03585556. Accessed 6 July 2020.

[94] AKST4290: an oral small molecule CCR3 antagonist. 2019. https://ois.net/wp-content/uploads/2019/07/Alkahest-FINAL-7-22-19.pdf.

[95] Chaney P. Once-daily topical anti-VEGF eye drop for wet AMD and other neovascular eye disease. Ophthamology Innovation Summit.

[96] Danis R, McLaughlin MM, Tolentino M (2014). Pazopanib eye drops: a randomised trial in neovascular age-related macular degeneration. Br J Ophthalmol.

[97] Csaky KG, Dugel PU, Pierce AJ (2015). Clinical evaluation of pazopanib eye drops versus ranibizumab intravitreal injections in subjects with neovascular age-related macular degeneration. Ophthalmology.

[98] Squalamine eye drop fails in wet AMD. No benefit seen when combined with Lucentis. 2018. https://www.retinalphysician.com/issues/2018/january-2018/squalamine-eye-drop-fails-in-wet-amd.

[99] Joussen AM, Wolf S, Kaiser PK, et al. A combined phase 2a/b study of the efficacy, safety, and tolerability of repeated topical doses of regorafenib eye drops in treatment-naïve patients with neovascular age-related macular degeneration (nAMD). Investig Ophthalmol Vis Sci. 2016;57.

[100] First-in-human study of LHA510 in elderly subjects and patients with age-related macular degeneration. 2016. https://clinicaltrials.gov/ct2/show/NCT02076919.

